# Field-induced assembly of colloidal ellipsoids into well-defined microtubules

**DOI:** 10.1038/ncomms6516

**Published:** 2014-11-20

**Authors:** Jérôme J. Crassous, Adriana M. Mihut, Erik Wernersson, Patrick Pfleiderer, Jan Vermant, Per Linse, Peter Schurtenberger

**Affiliations:** 1 Physical Chemistry, Department of Chemistry, Lund University, SE-22100 Lund, Sweden; 2 Department of Chemical Engineering, KU Leuven, University of Leuven, B-3001 Heverlee, Belgium; 3 Department of Physics, University of Konstanz, D-78457 Konstanz, Germany; 4 Department of Materials, ETH Zürich, CH-8093 Zurich, Switzerland

## Abstract

Current theoretical attempts to understand the reversible formation of stable microtubules and virus shells are generally based on shape-specific building blocks or monomers, where the local curvature of the resulting structure is explicitly built-in via the monomer geometry. Here we demonstrate that even simple ellipsoidal colloids can reversibly self-assemble into regular tubular structures when subjected to an alternating electric field. Supported by model calculations, we discuss the combined effects of anisotropic shape and field-induced dipolar interactions on the reversible formation of self-assembled structures. Our observations show that the formation of tubular structures through self-assembly requires much less geometrical and interaction specificity than previously thought, and advance our current understanding of the minimal requirements for self-assembly into regular virus-like structures.

## Abstract (web summary)

Building artificial nanostructures via self-assembly normally starts with shape-specific building blocks of colloidal particles or monomers. Here, Crassous *et al.* challenge this convention by showing that simple ellipsoids can form sheet-like or tubular structures in an alternating electric field.

## Graphical abstract

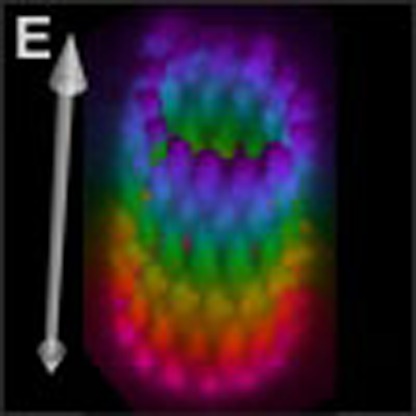

The idea of creating complex artificial nanostructures through simple self-assembly of small molecules, macromolecules or colloids may seem like an overly simplistic approach to building novel nanomaterials and devices^1^,^2^,^3^. Yet nature has excelled in fabricating complex functional structures such as microtubules and virus shells through self-assembly^4^,^5^,^6^. It is thus tempting to use a bio-inspired approach and exploit analogies to biological microtubule and virus assembly to make similar artificial structures from colloidal building blocks. However, this requires in-depth understanding of the underlying biological assembly processes.

Current theoretical attempts to understand the subtle interplay between the interactions that lead to the reversible formation of stable microtubules and virus shells have already advanced our knowledge significantly^5^,^7^,^8^,^9^,^10^. These attempts are, however, all based on shape-specific building blocks or monomers, where the local curvature of the resulting structure is explicitly built-in via the monomer geometry^9^,^10^. Here we now report on a new colloidal self-assembly mechanism for the formation of microtubule- and virus-shell like structures that challenges these currently accepted geometrical requirements. We describe the unexpected observation that prolate ellipsoidal colloids self-assemble into well-defined and highly regular tubular structures in the presence of an AC electric field, and discuss these findings in view of the current understanding of the minimal requirements for biological and synthetic virus-like nanostructures.

Our work also has to be seen in the context of the current attempt to combine so-called bottom-up and top-down approaches in nanotechnology; directed self-assembly (DSA) has moved into the focus of the soft-matter and nanotechnology community. DSA employs the basic principles of self-assembly through carefully chosen building blocks, but can be made more versatile by applying external electric, magnetic or shear fields^11^,^12^. DSA not only facilitates self-assembly, but often leads to the reversible formation of novel ordered or amorphous structures^12^,^13^,^14^. While most published DSA studies have focused on spherical particles, recent work with anisotropic particles has shown that the particle shape can have a strong and often surprising effect on their field-induced spatial organization^15^,^16^,^17^.

## Results

### Electric field-directed assembly of colloidal ellipsoids

**Figure 1:**
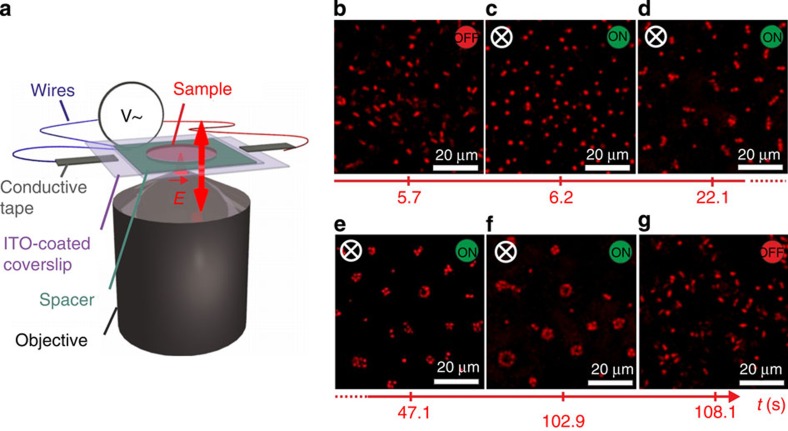
Temporal evolution of the reversible field-directed tubular self-assembly. (**a**) Schematic representation of the experimental set-up with an electric AC field along the *z-*direction. (**b**–**g**) CLSM *xy*-images illustrating the time evolution of reversible self-assembly of ellipsoidal particles (aspect ratio *ρ*=3.3, concentration 1 wt%, volume fraction *φ*≈0.04) before, during and after application of an alternating field *E*=167 kV m^−1^ at *f*=160 kHz (see also Supplementary Movie 1). Observations were made 10 μm away from the cover slide to minimize wall effects.

We investigated field-directed assembly as a function of particle anisotropy and employed composite microgels with prolate ellipsoidal shapes of aspect ratios *ρ*=2.3, 2.7, 3.3 and 8.8, dispersed in deionized water at 1 wt% (see Methods, Supplementary Fig. 1 and Supplementary Table 1 for details on synthesis and particle characterization)^18^. The samples were contained between two conducting glass slides coated with indium tin oxide (ITO) and separated by a 120-μm spacer as shown in Fig. 1a. The application of an AC voltage across the two slides produced a spatially uniform electric field. The field polarizes the colloids predominantly by the conduction of counterions within the microgel layer. This results in a time-varying polarization according to the Maxwell-Wagner-O’Konski mechanism, which is analogous to polarization due to a contrast in dielectric properties but also accounts for the contrast in conductivity^19^.

The chosen field frequency *f*=160 kHz corresponds to a situation where the particles rapidly align and self-assemble in response to the field without exhibiting any apparent drift due to electrode polarization effects that dominate at much lower frequencies (see Methods and Supplementary Fig. 2 for a more detailed discussion on the frequency dependence and Supplementary Fig. 3 for more information on the effect of ionic strength)^20^.

The response of the system was visualized *in situ* by confocal laser scanning microscopy (CLSM) in image planes perpendicular (*xy*) and parallel (*xz*) to the field. Figure 1b–g shows a series of 2D *xy*-images illustrating the temporal evolution of the field-induced self-assembly for *ρ*=3.3 (see Supplementary Movie 1). Without field, the colloidal particles are homogeneously distributed and their orientation is randomized by Brownian motion. When the field is switched on at sufficient field strength, the particles swiftly align along the field as seen from the circular particle cross-sections. After a few seconds, the particles build up string-like aggregates with a length of a few particles, which extend parallel to the field. Their linear cross-section indicates a sheet-like structure. The number of particles in the sheet cross-sections increases until they close and adopt a tubular structure. This tubular self-assembly is reversible; the particles quickly re-disperse after the field is turned off.

**Figure 2:**
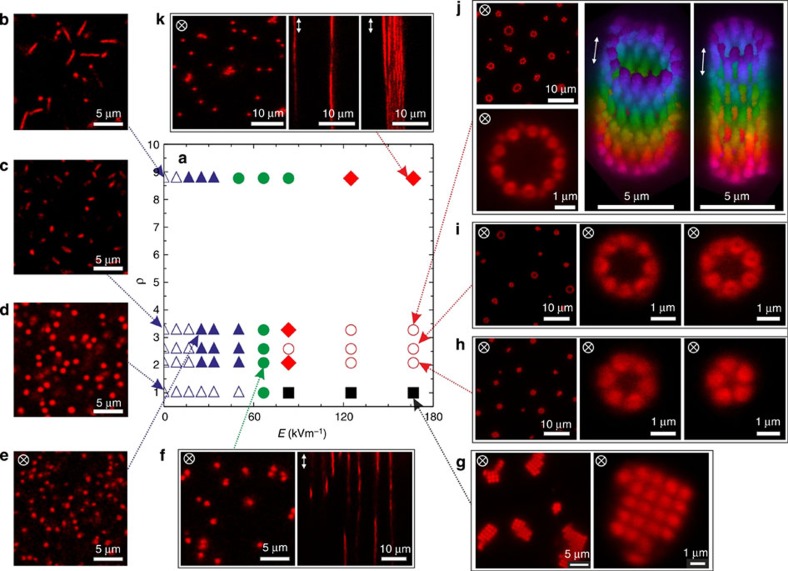
Overview on field-directed self-assembly. (**a**) Experimental state diagram of 1 wt% dispersions as a function of field strength *E* ((**b**–**d**) *E*=0 kV m^−1^, (**e**) 25 kV m^−1^, (**f**) 65 kV m^−1^ and (**g**–**k**) 167 kV m^−1^) and aspect ratio *ρ* at a frequency *f*=160 kHz, displaying (**b**–**d**) an isotropic particle fluid (open blue triangles), (**e**) an aligned particle fluid (solid blue triangles), (**f**) a string fluid (solid green circles), (**g**) ordered BCT structures (solid black squares), (**h**–**j**) regular tubular structures (empty red circles) and (**k**) amorphous solid filaments (red diamonds). Also shown are typical *xy*- and *xz*-images, and two 3D-reconstructions of tubular structures for *ρ*=3.3 and *E*=167 kV m^−1^ (colour coded according to the position along the *z*- direction).

The nature of the self-assembled structures varies with the applied field and particle anisotropy. A state diagram as a function of field strength *E* and aspect ratio *ρ* is given in Fig. 2, which also shows a selection of 2D snapshots and 3D reconstructions illustrating the different structures observed. Spherical particles (*ρ*=1) initially form strings that then assemble into ordered domains with body-centered tetragonal (BCT) symmetry at larger *E,* in agreement with previous reports for other spherical colloids^14^,^21^,^22^,^23^,^24^. Ellipsoidal particles (*ρ*>1) are aligned at *E*≈25 kV m^−1^. This field is sufficient to overcome Brownian rotation of the particles and corresponds to an energy of the aligned polarized particles in the external field similar to thermal energy, or about 1 *k*_B_*T*, where *k*_B_ is the Boltzman constant and *T* the temperature. For 2.2≤*ρ*≤3.3, with increasing *E* strings form across the full electrode gap, which then assemble into well-defined tubular structures. As illustrated by the two 3D reconstructions in Fig. 2, the tubes have a highly regular structure with a circular cross-section, single-particle wall and a periodic arrangement of the aligned particles. Once formed, neither a further evolution of the tubular cross-section with time nor any indication of monomer exchange could be observed. The average number of particles in the cross-section and their polydispersity increases with increasing *ρ* from 5±1 at *ρ*=2.1, 6±2 at *ρ*=2.7 and to 9±3 at *ρ*=3.3. For *ρ*=8.8 no tubes form, and we observe a coexistence of a few disordered string-like filaments with individual particle strings.

### Computer simulations

We used computer simulations to demonstrate that these morphologies are a direct consequence of the combination of a prolate ellipsoidal shape and a polarizing external field. We considered a simple model of uniformly polarized ellipsoids. Apart from the interaction due to the polarization, the particles are repulsive and we represent them by hard prolate spheroids of uniform size and shape. We expect this to be sufficient to capture the qualitative features of the system, but caution that explicit consideration of (screened) interactions between charges in the gel layers may be necessary for quantitative work (see Methods and Supplementary Fig. 3 for more information on the effect of ionic strength). The particles are aligned with their long axis in the *z*-direction and have a polarization that approximately corresponds to a uniform external field. This polarization is represented by a pair of point charges of equal magnitude *q* and opposite sign, placed symmetrically on the long axis at a distance *d* from the centre. The value of *d* was chosen according to


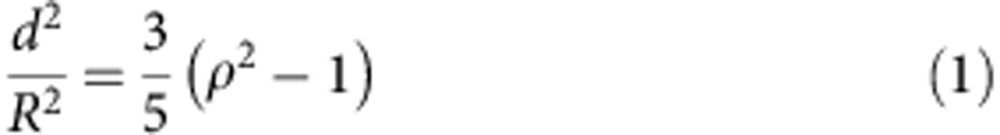


where *R* is the length of the short semi-axis, which reproduces the ratio of the two lowest non-vanishing multipole moments^25^.

**Figure 3:**
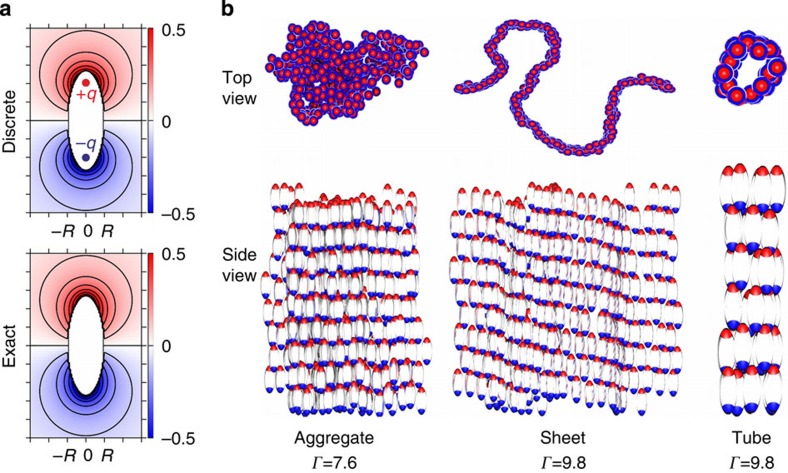
Computer simulations of field-directed self-assembly. (**a**) Dimensionless electrostatic potential Φ^*^=(4π*ε*_*m*_*R*^*2*^*/*2*qd*)Φ from a discrete charge representation of a polarized ellipsoid and its exact potential. (**b**) Self-assembled structures obtained from simulation: compact aggregate obtained at *Γ*=7.6, sheet-like aggregate obtained at *Γ*=9.8 and tubular aggregate obtained at *Γ*=9.8 and a lower number density.

As the experiment is carried out at a frequency above the characteristic relaxation frequency of the surrounding medium, the free charges in the medium cannot respond appreciably to the dynamic polarization of the particle. Therefore, no screening is included in the model. The electrostatic interaction energy can be written in dimensionless form as


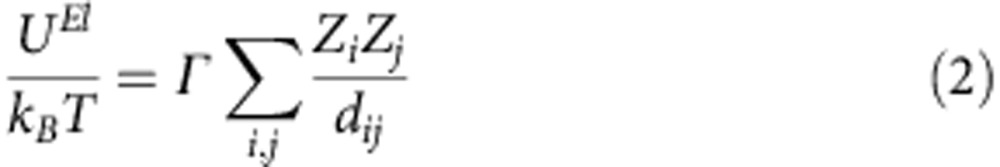


where the sum is over all pairs of charged sites *i* and *j* located on different particles and *Z*_*i*_ (*Z*_*j*_) gives the sign of the charge of site *i* (*j*) as 1 or −1. The reduced distance


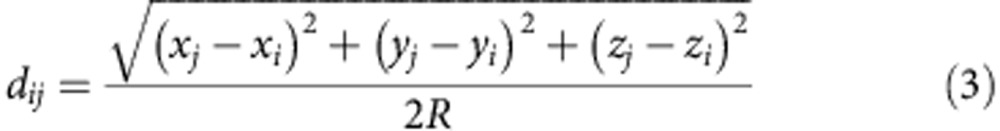


gives the distance between sites relative to the minimum particle centre-to-centre distance. The coupling parameter *Γ*=*q*^*2*^/(8*πε*_*m*_*Rk*_*B*_*T*), where *ε*_*m*_ is the medium permittivity, completely determines the strength of the electrostatic interactions. This discrete-charge representation closely approximates the exact solution for the electrostatic potential *Φ* (Fig. 3a)^26^.

We performed Metropolis Monte Carlo (MC) simulations to investigate the equilibrium properties of the model for varying *Γ*. Simulations were performed for particles with *ρ*=2.7 at a volume fraction *φ*=0.06 under periodic boundary conditions, which have the effect that a cluster that spans the simulation box is effectively infinite. A disordered aggregate formed for *Γ*≈5–8, and an ordered sheet that spans the system in two directions formed for *Γ*>8 (Fig. 3b). The poles of particles in adjacent rows of the sheet are interwoven to form arrays of alternating charges. Tubes were formed in simulations with *φ*=0.015, for which a sheet could not span the system in the *xy*-direction, strongly suggesting that the tube formation requires finite aggregate size. Representing the particle polarization by an ideal dipole did not produce any of these morphologies.

**Figure 4:**
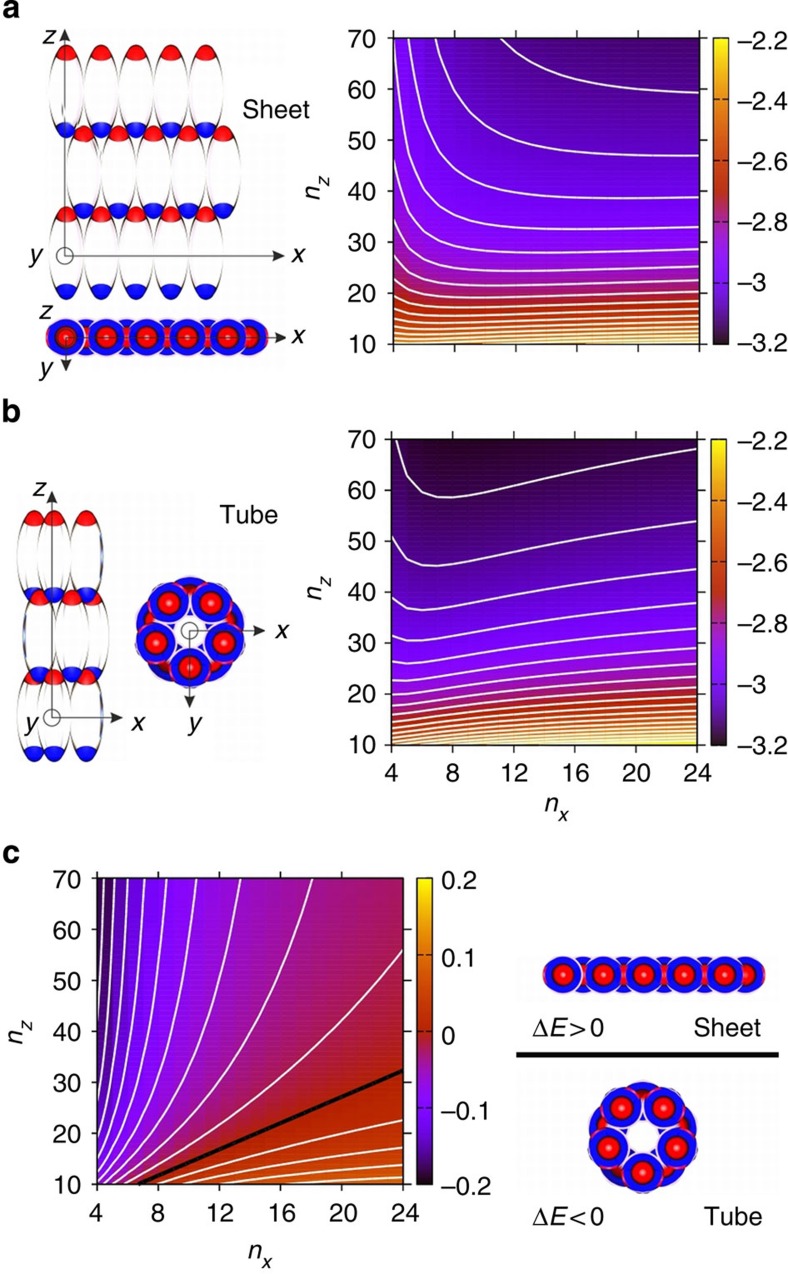
Minimum energy calculations. Schematic illustration and minimum reduced energy per particle (**a**) *E*_sheet_ for a sheet, (**b**) *E*_tube_ for a tube and (**c**) their energy difference Δ*E=E*_tube_*−E*_sheet_ as a function of the number of particles *n*_*z*_ and *n*_*x*_ parallel and perpendicular to the particle long axis. Energies are given relative to the minimum energy per particle in a dimer. The contour spacing is 0.05 in **a** and **b** and 0.02 in **c**.

To improve our understanding of the stability of the aggregates formed, we computed the energy per particle of idealized tubes and sheets in the minimum-energy configurations of these morphologies (see Fig. 4a,b and Methods). Minimized energies per particle are given as a function of *n*_*x*_ particles perpendicular and *n*_*z*_ particles parallel to the field axis. Both types of structures display a decreasing energy with increasing *n*_*z*_ and energy minima at increasing *n*_*x*_, the minima appearing at small *n*_*z*_ for a sheet and at large *n*_*z*_ for a tube. A tube attains lower energy than a sheet at large *n*_*z*_ and/or small *n*_*x*_ (Fig. 4c). These observations are largely explained by edge effects. An *xy*-edge contains uncompensated charges of the same sign, whereas there is an alternation in sign between adjacent charges along a *z*-edge. In a tube, a *z*-edge is eliminated and the associated energy cost is avoided. The energetic cost associated with *xy-*edges is, however, greater as the like-signed uncompensated charges are closer together. If *n*_*z*_ is large relative to *n*_*x*_, tubes will nevertheless be energetically favourable. Thus, this simple model shows that tubes are more stable than sheets already at a relative short tube length.

## Discussion

Our discovery that prolate ellipsoids form tubular aggregates on polarization, and alignment by an electric field shows that tubular self-assembly can result from simpler interactions than previously thought. The prolate shape makes the induced poles of the particles fit together in a zipper-like way to form ordered, two-dimensional sheets. Two competing effects of the uncompensated charge on the sheet edges make them close to form hollow tubes. This electrostatic mechanism is fundamentally different from the highly specific interactions that give rise to other tubular structures, for example, virus shells.

Most previously published theoretical and simulation studies on the formation of biological or artificial tubules and tubular virus shells such as tobacco mosaic viruses (TMVs) start from a monomer geometry that implicitly builds in a preference for a local curvature, and the interactions between the monomers primarily control the growth and stability of the self-assembled structure^9^,^10^. It is in fact interesting to compare the state diagram of our system (Fig. 2) with the different structures found in computer simulations of artificial microtubule formation^10^. These authors used wedge-shaped monomers with a geometrical preference for tube formation in their attempt to develop a minimal model for tubular self-assembly. They also encountered filaments or large sheets on variations of the lateral and vertical bonding interaction, similar to our experimental observations. Our results now demonstrate that we can further relax the requirements on the monomer shape, and that similar self-assembly patterns can be achieved with simple ellipsoids when properly tailoring the anisotropic interactions.

It is clear that the design and production of future materials and devices for photonics, molecular electronics or drug delivery would enormously benefit if we were capable of self-assembling synthetic nanostructures with the precision and reliability found in biological self-assembly. The current trend in bio-inspired nano-technology and nano-engineering emphasizes the implementation of the concepts of specificity and directionality in colloidal self-assembly, and thus the development of complex ‘colloidal molecules’ with asymmetric shapes and a finite number of specific and directional binding sites to achieve this^27^,^28^,^29^. Our combined experimental and simulation study now shows that complex shapes such as regular tubules can also be fabricated through directed self-assembly of very simple colloidal building blocks by a previously not considered electrostatic mechanism based on a delicate balance of anisotropic electrostatic interactions only. We believe that our results will contribute to the development of rules and minimal requirements for creating complex structures through self-assembly. They will thus not only provide new insight into possible mechanisms underlying virus shell and biological microtubule formation, but allow us to make a next step towards the ambitious goal of building synthetic nanostructures through a selective bio-inspired self-assembly process.

## Methods

### Composite microgels synthesis

*N*-isopropylmethacrylamide (NIPMAm; Aldrich), *N*,*N*′-methylenebisacrylamide (BIS; Fluka), sodium dodecyl sulfate (SDS; Fluka), methacryloxyethyl thiocarbamoyl rhodamine B (MRB) and potassium peroxodisulfate (KPS; Fluka) were used as received. Styrene (BASF) was purified on an Al_2_O_3_ column before use. Water was purified using reverse osmosis (MilliRO; Millipore) and ion exchange (MilliQ; Millipore). The poly(styrene)/poly(*N*-isopropylmethacrylamide) (PS/PNIPMAm) core-shell particles were synthesized in a two-step reaction as described in a previous study^18^. The same core particles were used for the present synthesis and employed as seeds for radical polymerization of the PNIPMAm shell in presence of the *N*,*N*′- methylenebisacrylamide (BIS) crosslinker (5 mol%). The only difference with the former recipe is the addition of a small amount MRB dye solution (162 μg dissolved in water) that was implemented to covalently label the particle shell.

### Mechanical deformation method

The anisotropic particles were obtained using the mechanical stretching method for PS lattices developed by Ho *et al.*^30^ In a first step, hybrid PVA polymeric films containing the core-shell particles were prepared following the procedure described in more details elsewhere^31^. Here, the core-shell particles (10 ml 4.5 wt% suspension) were first dispersed in an aqueous saturated polyvinyl alcohol solution (505 g, PVA; 40–88 from Fluka, *M*_w_≈205,000 g mol^–1^). After homogenization, the mixture was poured on a large glass tray (1,350 cm^2^) and allowed to dry into a thin film (≈150 μm thick) at room temperature for 4 days. The film was recovered and cut into 3-cm wide stripes. These stripes were then held between two clamps in a custom-made automatized uniaxial deformation set-up and stretched in a silicon oil bath maintained at 145 °C after an equilibration time of 5 min. This temperature, above the glass transition temperature *T*_g_ of the polystyrene^32^, ensures that the PS core plastically deforms. The stripes were stretched at different draw ratios corresponding to deformations *γ=Δl/l*_0_ (where *Δl* is the difference in length before and after stretching, and *l*_0_ the initial length of the film) of 50, 75, 100 and 400%, cooled down to room temperature and sorted according to their local deformation *γ*, accessible via a grid initially sketched at the surface of the film. To remove traces of oil, the films were further washed six times in isopropanol (45 min stirring, room temperature). The dissolution step first proceeds by stirring the films in isopropanol/MilliQ mixture (3:7 v/v) for 12 h at room temperature and then by heating the suspension at 75 °C for 45 min. The suspension was then centrifuged (1 h at 5,000 r.p.m.) and redispersed by sonication for several minutes. The dissolution step was repeated three times in isopropanol/MilliQ mixture and one time in MilliQ. Finally, the complete removal of all the PVA was ensured by at least 10 cycles of centrifugation (10 min at 10,000 r.p.m.)/redispersion in MilliQ water. The suspensions were concentrated to a weight fraction of 1 wt%, and in most of the experiments deionized by adding ion exchange resins.

### Particle characterization

The aspect ratio of the particles shown in Supplementary Fig. 1 can be effectively adjusted as described in the mechanical deformation method. During the stretching procedure the particles adopt an ellipsoidal shape with the long axis along the stretching direction. The shape is retained after cooling to room temperature, that is, below the *T*_g_ of the PS core. The particles were then characterized using CLSM, TEM (see Supplementary Fig. 1), DLS and electrophoretic mobility measurements, and the results are summarized in Supplementary Table 1. The TEM analysis shows that the overall particle shape approaches that of a prolate ellipsoid, with an aspect ratio varying from *ρ*=2.5 to 12.8. The particles with the highest aspect ratio deviate from an ideal ellipsoidal shape and appear to have a more tortuous shape. As the microgel shell collapses during the drying step in the TEM preparation, TEM primarily provides information about the core size and shape. While TEM was performed on dried samples, the CLSM image analysis was made with particles suspensions, monitoring particles that adsorbed at the water/glass interface. These experiments thus characterize the overall size and shape in the fully swollen state, and they provided a smaller variation of the aspect ratio from 2.1 to 8.8. We have subsequently used this estimate of *ρ* in the determination of the state diagram shown in Fig. 2. In addition, the thickness of the PNIPMAm shell along the short, *t*_*a*_, and long, *t*_*b*_, axis was evaluated based on the size of the core particles *R*=267 nm as determined by TEM in our former study^18^. This study has shown that in the collapsed state both core and core-shell have the same aspect ratio, which allows us to calculate the size of the PS core for both main full axes, 2*a*_*c*_ and 2*b*_*c*_, and to estimate the thickness of the shell from the difference with the CLSM results assuming an isochoric deformation.

The polydispersity of the two main full axes (*2a* for the long and *2b* for the short axis, respectively) is ≪10% as obtained from the TEM and CLSM images. From the variations of *2a* and *2b* during the elongation process obtained from both TEM and CLSM, we conclude that the stretching procedure corresponds to an almost isochoric transformation. DLS revealed that the particles are well-dispersed (see Supplementary Table 1) and the temperature dependence of *R*_h_ confirms that the thermoresponsiveness of all the particles was preserved in the stretching process. In addition, the particles are slightly negatively charged as evidenced by measurements of their electrophoretic mobility, *μ*, at low ionic strength, as a consequence of the presence of residual surfactant molecules and initiator fragments. The overall effective charge of the different particles was approximated as *Q*_eff_=*6πηR*_*h*_*μ*. The resulting average charge from all the different aspect ratios at 20 °C was estimated as 1,900±300 *e*^−^ with *e*^−^=−1.6 × 10^−19^ C.

### Dynamic light scattering

Dynamic light scattering (DLS) measurements were carried out using a light scattering goniometer instrument from LS Instruments equipped with a He-Ne laser operating at *λ*=632.8 nm. The temperature was controlled with an accuracy of 0.1 °C. The samples were highly diluted to 0.01 wt% to prevent interaction effects and multiple scattering. DLS measurements were performed at scattering angles of 45, 60 and 75° for temperatures ranging between 20.0 and 50.0 °C.

### Transmission electron microscopy

Samples for conventional transmission electron microscopy (TEM) were prepared by drop casting dilute (about 0.1 wt%) dispersions on a 300 mesh carbon-coated copper grid placed on a paper filter at room temperature. The micrographs were taken with a TEM-CM100 (Philips) operating at an acceleration voltage of 80 keV.

### Electrophoretic mobility

To estimate the overall effective charge of our particles, we measured the electrophoretic mobility of 0.01 wt% deionized dispersions. The measurements were performed on a Zetasizer Nano (Malvern) with strict control of the temperature set by Peltier elements at ±0.1 °C. The instrument was operated with a 4-mW 633-nm He-Ne laser.

### CLSM experiments

All electric field studies were carried out using an inverted confocal laser scanning microscope (CLSM) (Leica DMI6000) with an SP5 tandem scanner in the resonant mode using a × 100 immersion objective with a numerical aperture of 1.4. The CLSM is mounted in a thermostated enclosure, which enables temperature control with an accuracy of 0.2 °C. The microgel suspension (8 μl) was contained between the two conductive sides of the indium tin oxide (ITO)-coated microscope coverslips (SPI Supplies, Structure Probe Inc., USA; 30–60 Ω, 18 × 18 mm^2^) hermetically sealed by a 120-μm thick spacer with a 51-mm aperture (SecureSeal Imaging). Conductive tapes were then used to connect the ITO-coated coverslips to the electrodes of the frequency generator. In the current study, we worked at a constant temperature of 20 °C. Most of the measurements were made more than 10 μm away from the cover slide to minimize wall effects, except for investigations of the electrode polarization regime.

### Field frequency and ionic strength effect

To obtain additional information about the field-induced polarization of the particles, we conducted additional experiments at different values of the field frequency and the ionic strength of the dispersions. In the following, we first test the influence of the frequency *f* of the applied AC field at a field strength *E*=125 kV m^−1^ (see Supplementary Fig. 2). At lower frequencies *f*=1.6 and 16 kHz, electrode polarization effects dominate and the field becomes inhomogeneous as already observed in other studies^33^. As a result of the thus induced mass transport, the particles strongly drift towards the electrodes and self-assemble in 2D sheets perpendicularly to the surface of the electrodes. In this regime, the local number density in the vicinity of the electrode becomes significantly higher than in the bulk, and the self-assembly resembles the one discussed in Fig. 3b at high dipolar interactions. The field frequency *f*=160 kHz corresponds to the situation mostly discussed in the main article where the particles rapidly align and self-assemble in response to the field without exhibiting any apparent drift. At the highest frequency of *f*=1.6 MHz, however, the particles are no longer able to efficiently align in the field but are still dipolar and can partially align and form small aggregates.

We have also examined the effect of added salt. Supplementary Figure 3 summarizes the influence of the ionic strength on the field-induced self-assembly. Adjusting the salt concentration to 1 mM KCl results in a significant decrease of the particle polarizability, and the particles are still able to align but do not self-assemble into system-spanning string-like aggregates as was observed in fully de-ionized conditions. In addition, the field could not be maintained for >40 s at this ionic strength as the ITO coating started to electrolyze.

### Monte Carlo simulations

We performed MC simulations of a system of 384 particles with an aspect ratio of 2.7 with the box size chosen to correspond to a volume fraction *φ*≈0.06. This number density was close to the experimental one and was sufficient to enable formation of aggregates that span the system in two dimensions. Coupling parameters *Г* in the range 0 to 10 were considered. The system was periodic in all directions and electrostatic lattice sums were computed using the Ewald method. The MC Markov chain consisted of single-particle translations moved with a displacement parameter of one particle radius. Ellipsoid overlap was tested for using the overlap function from ref. 34. The simulation was started from a random configuration free of overlaps and was propagated for 10^7^ cycles using the MOLSIM software^35^. For these simulation conditions, compact and then sheet-like aggregates formed with increasing *Г* as described in the main text. Hollow fibres did not form for any *Г*. Such were found, however, in artificially restricted systems containing only 36 particles in a box with a size corresponding to *φ*=0.015, for which the available material allows the aggregates to span the system in one dimension only. Of ten statistically independent simulations for *Г*=9.8, all converged to a state with all particles aggregated into a hollow fibre that span the system in the *z*-direction.

We have chosen the model for the field-induced polarization mechanism described in the main text based on the observed frequency and ionic strength dependence. The frequency *f*=160 kHz at which the experiments were performed is about one order of magnitude above 1/*τ*_*q*_, where *τ*_*q*_ is the characteristic timescale of polarization, as estimated from conductivity measurements. As shown in Supplementary Fig. 2, polarization strongly depends on *f*, and for *f*=1.6 MHz the particles failed to respond appreciably to the field. The ionic strength also matters, and for 1 mM added salt the particles did align but not associate in the presence of a field at *f*=160 kHz (see Supplementary Fig. 3). As the charge relaxation frequency is increased ~100-fold by the added salt, this observation can be attributed to the screening of the particle interactions by the electrolyte. The self-assembly experiments are thus performed in the frequency range where the particles can respond to the field but the electrolyte medium cannot. We therefore expect that the electrostatic interactions between the polarized particles are not or only weakly screened by the electrolyte.

### Minimum energy calculations

We performed constrained energy minimization for sheet- and tube aggregates. To construct the sheet aggregates, particles were placed on a centred rectangular lattice with *n*_*z*_ particles in the direction of the particle long axis and *n*_*xy*_ particles along a perpendicular direction. The tube aggregates were constructed from the corresponding sheet aggregate by, effectively, distorting them into a cylinder such that neighbouring particles were in contact. According to Earnshaw’s theorem, the minimum of the potential must occur at particle contact. Thus, the lattice parameters are interdependent and can be characterized by a single parameter. We used the distance in *z* between adjacent particles, *l*_*z*_, for this purpose and performed one-dimensional energy minimizations in *l*_*z*_ for all combinations of *n*_*z*_ and *n*_*xy*_ such that 10≤*n*_*z*_≤70 and 4≤*n*_*z*_≤24 for sheet and tube aggregates.

## Author contributions

J.J.C., A.M.M. and P.S. designed the study; J.J.C. synthesized the particles and transformed them with the help of J.V. and P.P. into ellipsoids; J.J.C. and A.M.M. performed experimental research; E.W. and P.L. performed simulations; J.J.C., A.M.M. and E.W. analysed data; and J.J.C., A.M.M., E.W., P.L. and P.S. jointly wrote the paper.

## Additional information

**How to cite this article**: Crassous, J. J. *et al.* Field-induced assembly of colloidal ellipsoids into well-defined microtubules. *Nat. Commun.* 5:5516 doi: 10.1038/ncomms6516 (2014).

## Supplementary Material

Supplementary Information Supplementary Figures 1-3 and Supplementary Table 1

Supplementary Movie 1 Dipolar self-assembly of ellipsoidal composite microgels in an ac electric field.
