# Supplementary material for: Field-induced assembly of colloidal ellipsoids into well-defined microtubules
Source: Nat Commun. 2014 Nov 20;5:5516. doi: 10.1038/ncomms6516 (PMC4263160; doi:10.1038/ncomms6516)
Supplement: Supplementary Information — Supplementary Figures 1-3 and Supplementary Table 1 [file ncomms6516-s1.pdf]

Shape matters for a new colloidal self-assembly mechanism for synthetic microtubules

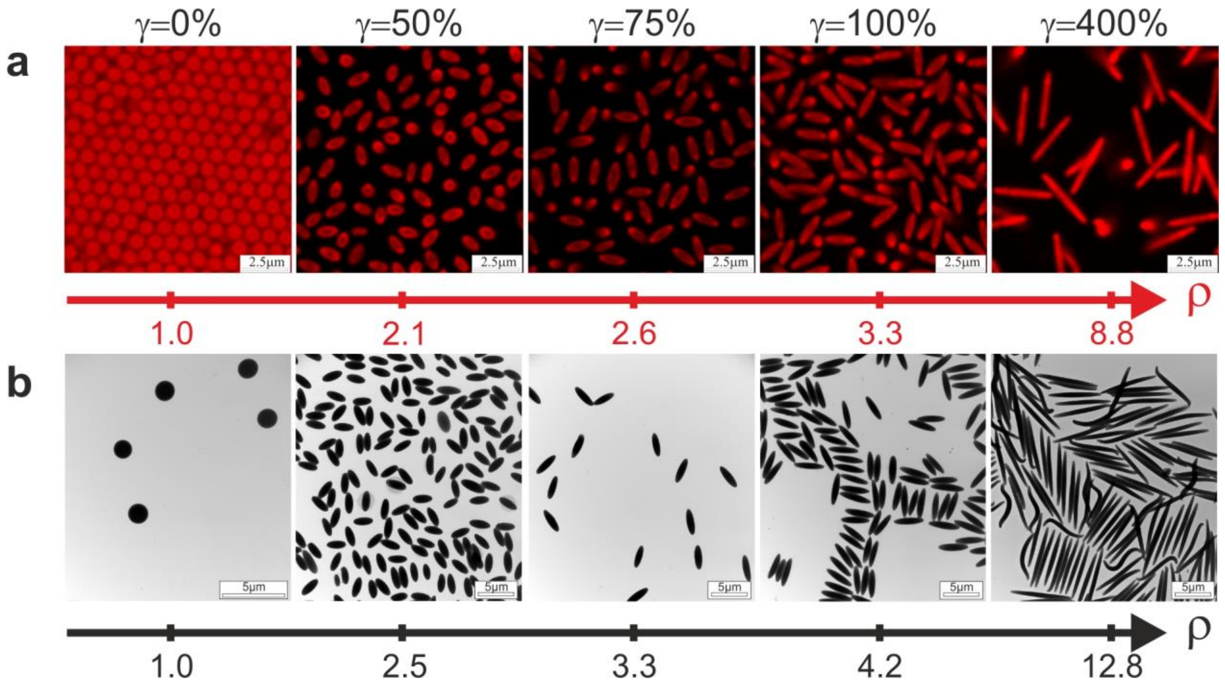

Supplementary Fig. 1 | Microscopy of composite ellipsoidal microgels synthesized with different aspect ratios. Microscopic characterization of the composite microgel morphologies obtained at different uniaxial deformation (*γ*). CLSM (**a**) and TEM micrographs (**b**) of the core-shell particles at the water/glass interface and in air on carbon-coated copper grids, respectively. The evolution of the aspect ratio (*ρ*) determined from the analysis of the different micrographs with the two different techniques is indicated as a function of the deformation.

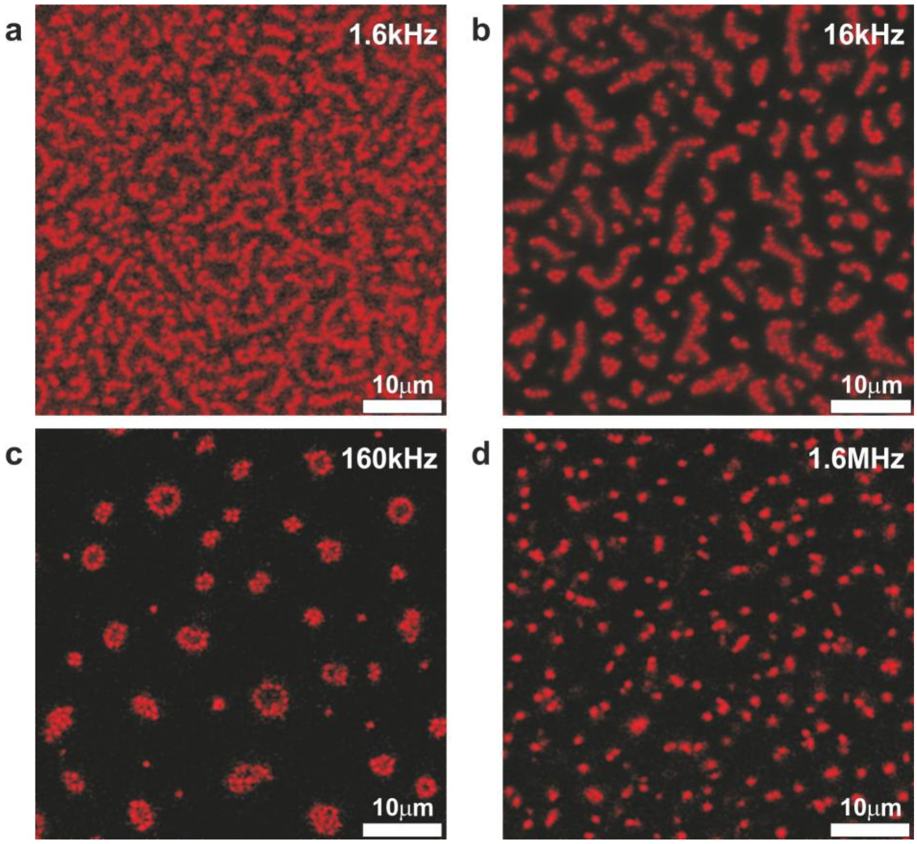

Supplementary Fig. 2 | Frequency dependence of the self-assembly. CLSM *xy*-images showing the assemblies formed by a 1 wt% ellipsoidal composite microgel dispersion (ρ = 3.3) in response to an applied AC electric field E = 125 kV m^{-1} as a function of frequency. The applied range of frequencies varied from f = 1.6 kHz to 1.6 MHz. Representative images show the formation of (**a**) solid worm-like lattices formed in the vicinity of the electrodes at f =1.6 kHz, (**b**) “dynamic” worm-like lattices which extend over 10 µm in the z-plane at f = 16 kHz, (**c**) tubular structures at f = 160 kHz, and (**d**) a partially aligned fluid at f = 1.6 MHz. All structures formed are fully reversible, and the colloidal dispersions relax to their initial fluid-like disordered state after the field is turned off.

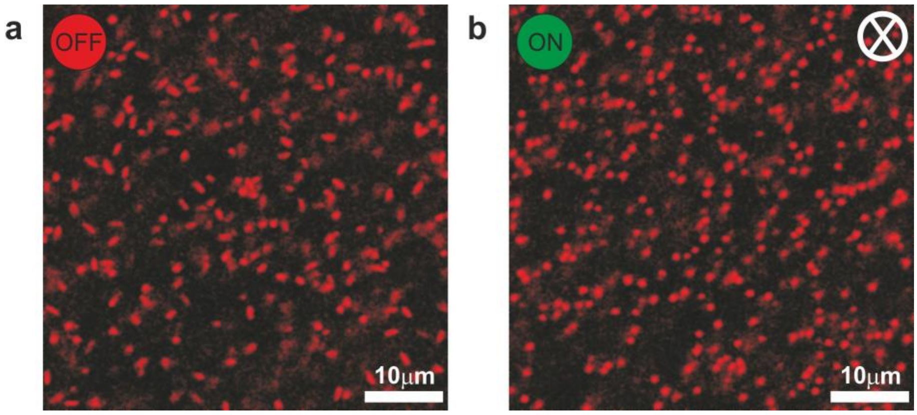

Supplementary Fig. 3 | Salt effect on the dipolar association. The experiment was performed on a 1 wt% ellipsoidal dispersion (ρ = 3.3) containing 1 mM KCl under an AC electric field E = 125 kV m^{-1} with a frequency, f = 160 kHz. The CLSM *xy*-images correspond to (**a**) randomly orientated ellipsoids (disordered particle fluid), (**b**) oriented ellipsoids with their major axes aligned in the field direction (aligned particle fluid).

Supplementary Table 1

γ	0%	50%	75%	100%	400%
$2a$ [nm] CLSM	824 ± 47	1276 ± 81	1528 ± 88	1770 ± 117	3308 ± 176
$2b$ [nm] CLSM	824 ± 47	614 ± 50	591 ± 38	542 ± 42	381 ± 26
ρ (CLSM)	1	2.1 ± 0.1	2.6 ± 0.2	3.3 ± 0.2	8.8 ± 0.7
$2a$ [nm] TEM	623 ± 31	1149 ± 58	1401 ± 71	1634 ± 90	3408 ± 267
$2b$ [nm] TEM	623 ± 31	459 ± 28	424 ± 22	392 ± 28	270 ± 32
ρ (TEM)	1	2.5 ± 0.1	3.3 ± 0.2	4.2 ± 0.3	12.8 ± 1.6
$2a_c$ [nm] TEM	534	986	1184	1388	2920
$2b_c$ [nm] TEM	534	393	359	331	228
ρ (TEM)	1	2.5	3.3	4.2	12.8
t_a [nm]	145	145	172	191	194
t_b [nm]	145	111	116	105	76
R_h at 20°C [nm] (DLS)	537	499	501	527	670
R_h at 50°C [nm] (DLS)	348	333	-	428	493
Q_{eff} at 20°C [e^-]	2104	1779	1601	1767	2148

Supplementary Table 1 | Summary of the main characterization results. Average dimensions and aspect ratios of the composite microgel particles stretched at a deformation of γ = 0%, 50%, 75%, 100% and 400% determined from statistical analysis of CLSM and TEM micrographs. The notations $2a$, $2b$ refer to the long and short full axis of the particles and $2a_c$, $2b_c$ to the long and short full axis of the PS core. t_a and t_b indicate the PNIPMAm shell thickness along the long and short axis, and ρ the determined aspect ratio. This table further includes the apparent hydrodynamic radius R_h measured by DLS at 20°C and 50°C and the effective charge of the different particles Q_{eff} estimated at 20°C from the combination of DLS and electrophoretic measurements of the different core-shell particles.
